# Analysis of the Neutralizing Activity of Antibodies Targeting Open or Closed SARS-CoV-2 Spike Protein Conformations

**DOI:** 10.3390/ijms23042078

**Published:** 2022-02-14

**Authors:** Gabriel Cia, Fabrizio Pucci, Marianne Rooman

**Affiliations:** 1 Computational Biology and Bioinformatics, Université Libre de Bruxelles, 1050 Brussels, Belgium; gabriel.cia@ulb.be (G.C.); fabrizio.pucci@ulb.be (F.P.); 2 Interuniversity Institute of Bioinformatics in Brussels, 1050 Brussels, Belgium

**Keywords:** COVID-19, immune response, spike protein variants, epitopes, binding affinity prediction, polyclonal plasma, escape fractions, immunoinformatics

## Abstract

SARS-CoV-2 infection elicits a polyclonal neutralizing antibody (nAb) response that primarily targets the spike protein, but it is still unclear which nAbs are immunodominant and what distinguishes them from subdominant nAbs. This information would however be crucial to predict the evolutionary trajectory of the virus and design future vaccines. To shed light on this issue, we gathered 83 structures of nAbs in complex with spike protein domains. We analyzed in silico the ability of these nAbs to bind the full spike protein trimer in open and closed conformations, and predicted the change in binding affinity of the most frequently observed spike protein variants in the circulating strains. This led us to define four nAb classes with distinct variant escape fractions. By comparing these fractions with those measured from plasma of infected patients, we showed that the class of nAbs that most contributes to the immune response is able to bind the spike protein in its closed conformation. Although this class of nAbs only partially inhibits the spike protein binding to the host’s angiotensin converting enzyme 2 (ACE2), it has been suggested to lock the closed pre-fusion spike protein conformation and therefore prevent its transition to an open state. Furthermore, comparison of our predictions with mRNA-1273 vaccinated patient plasma measurements suggests that spike proteins contained in vaccines elicit a different nAb class than the one elicited by natural SARS-CoV-2 infection and suggests the design of highly stable closed-form spike proteins as next-generation vaccine immunogens.

## 1. Introduction

A clear understanding of the molecular mechanisms used by the SARS-CoV-2 virus to escape the human immune system is urgently needed to overcome the current COVID-19 pandemic situation [1]. To that end, a lot of attention has recently been devoted to the understanding of the immune response induced by the virus, and in particular to the study of neutralizing antibodies (nAbs) that target viral surface proteins [2,3,4,5,6]. The current understanding is that the humoral immune response primarily elicits nAbs that target the receptor binding domain (RBD) of the spike protein [7], although nAbs targeting its N-terminal domain (NTD) have also been reported [8]; the rest of the spike protein surface is strongly glycosylated [9] and therefore shielded from immune recognition.

It is well known that the SARS-CoV-2 virus is able to invade host cells through the binding of its RBD to the angiotensin-converting enzyme 2 (ACE2) [10]. However, the presence of RBD-targeting nAbs puts the RBD under strong selective pressure. Missense mutations in the RBD that lead to immune evasion are likely to be selected [1,11], while mutations that abolish or reduce the binding affinity with ACE2 are not.

**Figure 1:**
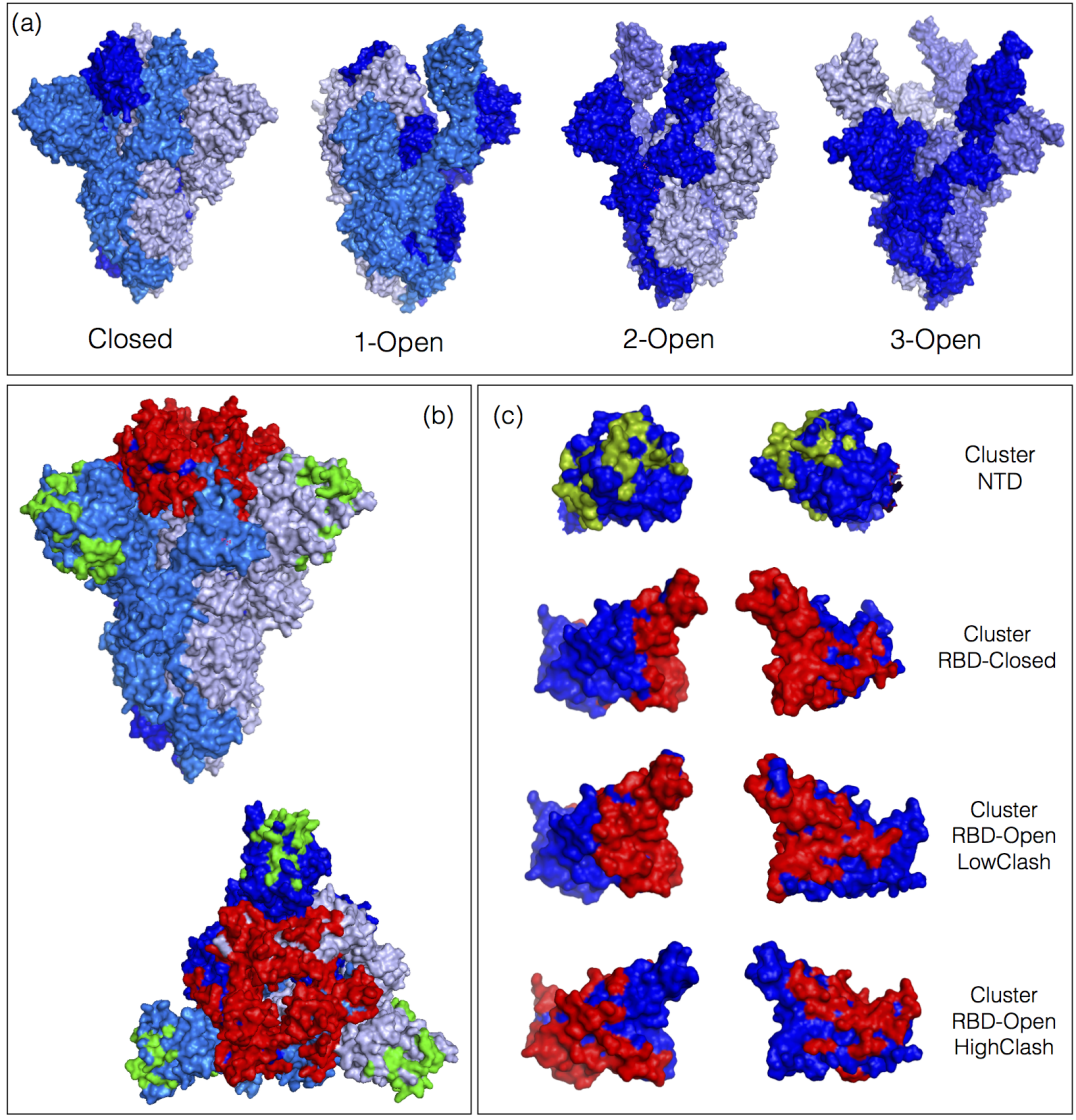
Spike protein conformations and epitopes. (**a**) 3D structure of the spike protein homotrimer in closed conformation (PDB code 6vxx), 1-open conformation (PDB code 6vyb), 2-open conformation (PDB code 6x2b) and 3-open conformation (PDB code 7cak). The three monomers are in different shades of blue. (**b**) Lateral and upper view of the 3D structure of the spike protein trimer in closed conformation (PDB code 6vxx). The ensemble of epitopes defined by the 83 neutralizing antibodies (nAbs) from the $D_{\mathrm{nAb}\text{-}\mathrm{spike}}$ dataset are colored in red and green according to whether they are situated on the receptor binding domain (RBD) or N-terminal domain (NTD). (**c**) NTD and RBD with the ensemble of epitope residues; the pairs of pictures are obtained by a 180° rotation around the vertical axis. The RBD epitopes are divided in three classes on the basis of the clash scores in the open and closed spike protein conformations (see Section 3.2).

It has already been observed that almost the entire RBD surface can be bound by nAbs [12,13] (see Figure 1), but it is still unclear how each individual nAb contributes to the neutralizing activity of the highly polyclonal immune response. The identification of which nAbs are dominant and which are subdominant is essential for analyzing and predicting the disease phenotype and the antigenic evolution. Indeed, mutations that reduce the binding affinity for dominant nAbs are more likely to lead to immune evasion than mutations affecting the affinity for subdominant nAbs.

Recent high-throughput deep mutagenesis experiments have led to the identification of several groups of nAbs that each target different epitope regions on the surface of the spike protein, and suggest that a single group of nAbs dominates the neutralizing activity of polyclonal plasma [14]. Indeed, the escape map of this nAbs group, consisting of RBD mutations causing immune evasion, strongly resembles the escape map observed in polyclonal plasma from convalescent patients.

In this study, we used a structural bioinformatics approach to extend the above described results [14]. More precisely, we analyzed a much larger dataset of nAbs, targeting both the RBD and NTD, and took into account the effect of the conformational changes undergone by the spike protein. Indeed, this protein can adopt two different conformations, an open (or up) and a closed (or down) conformation [15]. This conformational transition plays a key role in viral cell invasion, given that large parts of the RBD get exposed to the solvent in the open conformation, which allows its binding to ACE2 receptors located on the surface of host cells and therefore entry of the virus into the cells.

## 2. Methods

### 2.1. $D_{\mathrm{nAb}\text{-}\mathrm{spike}}$ Dataset Construction

We constructed a non-redundant dataset of nAbs using the coronavirus antibody database CoV-AbDab [16]. We only selected antibodies that target the SARS-CoV-2 spike protein and have an experimental 3-dimensional (3D) structure resolved by X-ray crystallography or cryo-electron microscopy with a resolution of at most 3.5 Å, available in the Protein DataBank (PDB) [17]. We splitted the structures containing multiple nAbs into multiple structure files, each containing a single nAb in complex with the spike protein. We got in this way 117 structures of reasonably well resolved nAb-spike protein complexes. Next, we clustered the nAbs using the sequence comparison and clustering program CD-hit [18], using a 99% sequence identity threshold.

This procedure led to a non-redundant dataset of 83 structures of nAb-spike protein complexes, called $D_{\mathrm{nAb}\text{-}\mathrm{spike}}$. Among these nAbs, 75 target the RBD and 8 the NTD. Note that they generally have very strong binding affinities, with a median $K_{d}$ = 3.5 nM. The list of PDB structures of the $D_{\mathrm{nAb}\text{-}\mathrm{spike}}$ dataset along with a list of their characteristics is available at https://github.com/3BioCompBio/SARS-CoV-2-Epitope (accessed on 5 January 2022).

### 2.2. Identification of Spike Protein Epitopes and ACE2 Binding Residues

The epitope of each nAb-spike protein complex from the $D_{\mathrm{nAb}\text{-}\mathrm{spike}}$ dataset was determined on the basis of the relative solvent accessibility (RSA) of each residue of the spike protein in its bound and unbound structure. The RSA of a residue is defined as the ratio between its solvent accessible surface area in a given structure and in an extended Gly-X-Gly tripeptide conformation and was computed via an in-house program [19]. The residues undergoing a change in RSA of at least 5% between bound and unbound states were defined as epitope residues: RSA${}_{\mathrm{unbound}}$ − RSA${}_{\mathrm{bound}}$ ≥ 5 %. Note that we defined epitopes considering only one spike protein chain; we thus made the hypothesis that nAbs only interact with a single monomer of the full spike protein trimer.

The unbound structure of the spike protein was taken to be the same as its structure bound to an nAb, thus neglecting possible conformational rearrangements upon binding. To check the validity of this approximation, we evaluated the conformational changes induced by nAb binding by calculating the full-atom root mean square deviation (RMSD) of each bound RBD or NTD structure in the $D_{\mathrm{nAb}\text{-}\mathrm{spike}}$ dataset with respect to the unbound spike protein structure 7cak [20]. The mean RMSD is found to be equal to 0.9 Å and 1.0 Å for RBD and NTD structures, respectively, which indicates that they do not undergo significant conformational changes upon nAb binding.

We used the same procedure to define the residues of ACE2 that bind to the RBD of the spike protein, using the PDB structure 6m0j, i.e., a change of at least 5% in RSA between the RBD bound to ACE2 and the RBD in unbound form. The RBD does not undergo significant conformational modifications upon ACE2 binding, as measured by an RMSD of 0.8 Å.

### 2.3. Estimation of Steric Clashes of nAbs with the Open/Closed Spike Protein Conformations

The biological unit of the spike protein is a homotrimer which can adopt different conformations: a closed conformation in which all three RBDs are in lying-down state and three open conformations in which one, two or three RBDs are in standing-up state, called for simplicity 1-open, 2-open and 3-open conformations; these 4 conformations are illustrated in Figure 1a. We considered the experimental structures, resolved by electron microscopy, of the closed, 1-open and 3-open states, with PDB codes are 6vxx [21], 6vyb [21] and 7cak [20], respectively.

Most structures of the $D_{\mathrm{nAb}\text{-}\mathrm{spike}}$ dataset contain a nAb in complex with either the RBD or NTD, but only rarely with the full spike protein. To determine which nAbs can bind to the open and closed spike protein conformations or only to the open conformation, we superimposed the structure of each entry of $D_{\mathrm{nAb}\text{-}\mathrm{spike}}$ onto the closed, 1-open and 3-open structures of the full spike protein trimer using PyMOL [22]. We then used the MolProbity webserver [23] to compute the steric clashes between each nAb and the trimeric spike protein in the three conformations. Steric clashes were defined between atoms of which the overlap of their van der Waals spheres was larger than 0.4 Å. We considered a nAb to be able to bind a given spike protein conformation if there were less than 500 steric clashes between atoms pairs of the two protein partners. This threshold value was chosen from the analysis of the clash score distribution, which shows an empty zone in the range of 500–1000 clashing atom pairs (see https://github.com/3BioCompBio/SARS-CoV-2-Epitope (accessed on 5 January 2022)). This observation suggests that the threshold of 500 steric clashes corresponds to the maximum amount of structural rearrangements that can occur when a nAb binds a spike protein conformation.

### 2.4. Prediction of nAb-spike Protein Binding Affinity Changes and Escape Fractions

In order to analyze the impact of spike protein variants on the neutralizing activity of nAbs, we computed the change in binding affinity upon mutation ($\Delta \Delta G_{b}$) of the nAb-spike protein complex, using our in-house tool BeAtMuSiC [24]. This program takes as input the 3D structure of the complex and predicts in a fast and accurate way the $\Delta \Delta G_{b}$ of single-site mutations. We considered the 83 complexes of the $D_{\mathrm{nAb}\text{-}\mathrm{spike}}$ dataset, and computed the effect of 40 different mutations in the spike protein on its affinity for the different nAbs. These 40 mutations were selected as the most frequent mutations observed in the circulating SARS-CoV-2 strains (collected from GISAID database [25] accessed in November 2021); we chose the 20 most frequent variants located in the RBD and the 20 most frequent variants situated in the NTD. Note that we considered that variants in the RBD have no effect on nAbs targeting the NTD and vice versa (i.e., $\Delta \Delta G_{b}$=0).

We also estimated the $\Delta \Delta G_{b}^{\mathrm{strain}}$ between the spike protein of the known SARS-CoV-2 strains and the 83 nAbs from the $D_{\mathrm{nAb}\text{-}\mathrm{spike}}$ dataset which are bound to either the RBD or the NTD. This was done by summing the $\Delta \Delta G_{b}^{i}$ computed separately for each point mutation *i* carried by the spike protein of the viral strain, and separately for each nAb complex. Note that, in doing that, we neglected possible epistatic effects due to interactions between mutated residues.

### 2.5. Measured Escape Map of Human Polyclonal Plasma

The effect of all possible single-site variants in the RBD on the neutralizing activity of the plasma taken from 10 COVID-19 patients [26] and from 23 healthy individuals vaccinated with mRNA-1273 [27] have been experimentally characterized via deep mutagenesis. The measured escape fractions of variants were shown to be positively correlated with the ability of the viral strain to escape the polyclonal antibody response of a patient. Given that the escape fractions are available at multiple time points for each patient, we considered the average escape fraction over time.

To estimate the total escape ability of circulating viral strains from the plasma of COVID-19 infected patients or vaccinated individuals, we summed the escape fractions of all single-site variants carried by the spike protein that have been experimentally measured by deep mutagenesis [26], thus again neglecting epistatic effects. Note that, since experimental escape fractions are only available for variants in the RBD, the contribution of NTD variants and their impact on the neutralizing activity of nAbs were completely neglected.

## 3. Results

### 3.1. Epitopes in the $D_{\mathrm{nAb}\text{-}\mathrm{spike}}$ Dataset

We set up the $D_{\mathrm{nAb}\text{-}\mathrm{spike}}$ dataset containing 83 non-redundant and well-resolved 3D structures of nAb-RBD and nAb-NTD complexes (Section 2.1) and analyzed them in silico. As a first step, we identified the epitopes in each of the nAb-spike protein complexes (Section 2.2). This analysis yielded the well known result that the immune response against SARS-CoV-2 mostly involves nAbs that target the RBD of the spike protein [7], but that some nAbs target the NTD [8]. The rest of the spike protein is strongly glycosylated and does not seem to contain epitopes [9].

Moreover, we found that almost all the surface residues of the RBD are part of the epitope of at least one of the nAb-RBD complexes from the $D_{\mathrm{nAb}\text{-}\mathrm{spike}}$ dataset, as shown in Figure 1c. In contrast, many surface residues of the NTD are not part of any epitopes. It is difficult to conclude at this stage whether the latter observation is a characteristic of the NTD or just a consequence of the small number of nAb-NTD complexes in the $D_{\mathrm{nAb}\text{-}\mathrm{spike}}$ dataset.

### 3.2. Classes of nAbs Based on their Ability to Bind Open or Closed Spike Protein Conformations

The spike protein has a homotrimeric quaternary structure which can adopt several conformations characterized by the RBDs being in either lying-down or standing-up state. We call these conformations closed, 1-open, 2-open and 3-open, as defined in Section 2.3 and illustrated in Figure 1a. The question we addressed here is whether the nAbs can bind to the spike protein in these different conformations. Indeed, the $D_{\mathrm{nAb}\text{-}\mathrm{spike}}$ dataset mostly contains nAb-RBD and nAb-NTD complexes, which makes it impossible to directly determine whether the nAbs are able to bind the closed conformation of the spike protein trimer or only the open conformations.

**Figure 2:**
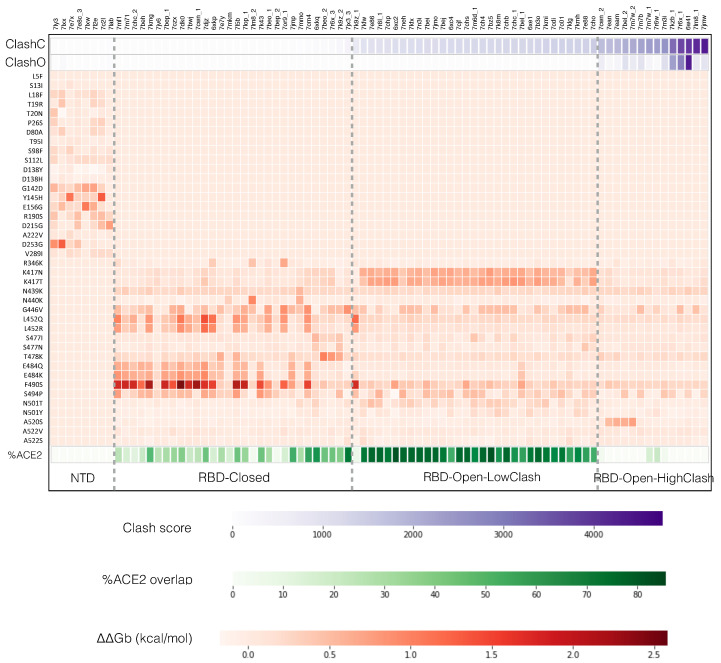
Heatmap in which each column corresponds to one of the 83 neutralizing antibodies (nAbs)-spike protein structures from the $D_{\mathrm{nAb}\text{-}\mathrm{spike}}$ dataset, indicated by their PDB code and ordered according to their clash score in the closed conformation. Row 1 and 2: clash score when the nAb-spike protein complex is superimposed onto the full trimeric spike protein structure in closed (ClashC) and 1-open (ClashO) conformations, respectively; the scale shown below is in number of clashing atom pairs. Rows 3-42: Predicted change in binding affinity upon mutation (ΔΔ*G_b_*), for the 40 variants studied, specified in ordinate; the free energy scale in kcal/mol is given below. Row 45: overlap between the epitope and the angiotensin-converting enzyme 2 (ACE2) binding region on the spike protein surface; the scale, in % of the ACE2 binding residues, is indicated below. The three vertical dashed lines indicate the four classes specified below.

To investigate this, we superimposed the nAb-RBD and nAb-NTD structures of the $D_{\mathrm{nAb}\text{-}\mathrm{spike}}$ dataset onto the different open and closed spike protein conformations and computed the associated steric clash scores, as described in Section 2.3. We also calculated the change in binding affinity $\Delta \Delta G_{b}$ of all these complexes caused by the 20 variants in the RBD and the 20 variants in the NTD which are the most frequently observed in circulating SARS-CoV-2 strains, in order to evaluate the impact of the variants on the neutralizing activity of the different nAbs, in other words to predict the escape map (see Section 2.4). The computed steric clash scores and the predicted escape map of each nAb are represented in Figure 2 in the form of a heatmap; the average clash scores per class are given in Table 1.

The simple ordering of the 83 nAb-spike protein complexes from the $D_{\mathrm{nAb}\text{-}\mathrm{spike}}$ dataset according to increasing clash score in the closed conformation reveals four distinct nAb escape patterns, without the need of any clustering, as shown in Figure 2. These patterns can readily be interpreted in terms of the ability of each of these four classes of nAbs to bind the different conformations of the spike protein trimer. The classes, depicted in Figure 1c, are the following:

- The **NTD class** contains nAbs that bind to the NTD. The clash score is in this case zero as this domain is always fully accessible, both in the closed, 1-open and 3-open conformations. Some nAb-NTD complexes of this group are not affected by the considered NTD variants, while other complexes are strongly destabilized, for example by the variants G142D, E156G, Y145H or D253G.
- The **RBD-Closed class** corresponds to nAbs that can bind the RBD in its closed conformation, given their low clash score with the full trimeric spike protein in closed conformation. Note that all these nAbs can also bind the open conformation. RBD variants that abolish the neutralizing activity of this class are mainly E484K, E484Q, F490S, L452R, L452Q and G446V.
- The **RBD-Open-LowClash** corresponds to nAbs that cannot bind the closed spike protein conformation but that do bind the open conformation, as shown by their intermediary clash score (500–2000) with the closed conformation and low clash score with the 1-open conformation. The escape pattern of this class strongly differs from that of the previous class: the neutralizing activity is reduced by RBD mutations such as K417N, K417T and G446V.
- The **RBD-Open-HighClash** contains nAbs that seem able to bind neither the closed nor the 1-open spike protein conformation, as demonstrated by their high clash score with both conformations. They can bind only the 2-open or 3-open conformations. Again, this class has a very different escape pattern, with little changes in neutralizing activity for the considered variants.

This classification into four groups extends and refines an analogous classification obtained from structural information on a few nAb-spike protein complexes [6].

### 3.3. Escaping Mechanisms and Key Residue Interactions

We analyzed in detail key residue-residue interactions at the interface between nAbs and the spike protein. We focused on cation-π interactions, π-π interactions, ionic interactions and hydrophobic contacts identified by the Protein Interactions Calculator (PIC) [28]. Variants that perturb these interactions have been observed in circulating SARS-CoV-2 strains (see Figure 2) and contribute to the immune evasion of the virus.

The residues most frequently involved in spike protein-nAb binding are listed in Table S1 and represented in Figure S2 of Supplementary Material. Their analysis shows that:

- Aromatic-involving interactions play a major role in nAb-spike protein binding, which is a general trend in antigen-antibody interfaces [19].
- Each class of nAbs targets different patterns of spike protein residues. However, there is a partial overlap between residues targeted by the RBD-Closed and RBD-Open-LowClash nAb classes, which results from the overlap between the two epitope regions, visible in Figure 1.
- Residues that are important for the binding of nAbs from the RBD-Closed class, such as E484, F490 and L452, are often mutated in the circulating strains (Figure 2). This indicates that the RBD-Closed epitope region is under strong selective pressure. The selective pressure on the RBD-Open-LowClash epitope region seems less strong as only K417 is found mutated in circulating strains.
- Residues that are important for spike protein binding to ACE2 (Table S2 and Figure S2) are bound by nAbs of both the RBD-Closed and RBD-Open-LowClash classes despite the fact that the epitope region from the latter overlaps more with the ACE2 binding region (see next section).

### 3.4. Contributions of the nAb Classes to the Immune Response

Recent experimental results comparing the escape map from plasma of convalescent COVID-19 patients and from a small set of nAbs suggest that not all nAbs contribute to the same extent to the polyclonal antibody response [14]. Rather, only one class of nAbs seems to be immunodominant and to drive viral evolution. We compared these experimental results with our extensive computational mutagenesis analyses.

We first computed the correlation between the measured escape fractions from ten COVID-19 patient plasma taken from [26] and the predicted change in affinity $\Delta \Delta G_{b}$ caused by the 20 most frequent RBD variants on the nAb-spike protein complexes contained in the $D_{\mathrm{nAb}\text{-}\mathrm{spike}}$ dataset, as described in Section 2.4. To get per-class $\Delta \Delta G_{b}$ values, we took the average over all nAb-spike protein complexes in the class.

**Figure 3:**
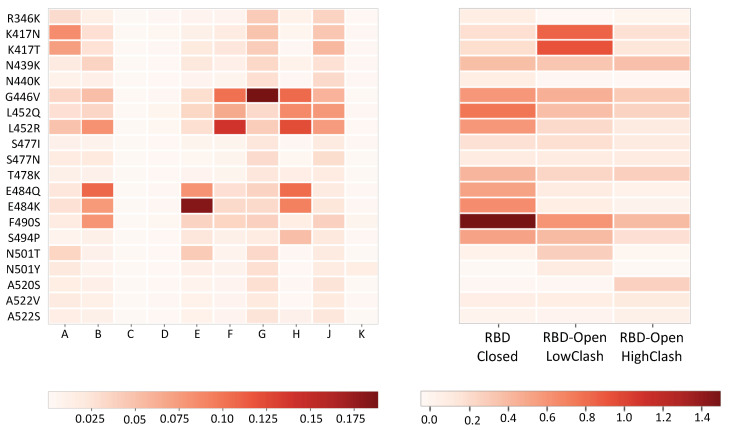
Heatmap in which each of the 20 rows corresponds to a variant in the receptor binding domain (RBD) (corresponding to rows 23–42 of Figure 2). Left panel: Variant escape fractions obtained from polyclonal plasma of ten COVID-19 patients noted A to K, taken from [26]. Right panel: predicted change in binding affinity upon mutation (ΔΔ*G_b_*) in kcal/mol for the same 20 variants, averaged over the nAb-spike protein complexes contained in the three RBD-targeting nAb classes. The escape fraction and $\Delta \Delta G_{b}$ scales are shown below.

We found that the RBD-Closed class is the only class that exhibits a statistically significant correlation of 0.64 (*p*-value = 0.003) with the measured escape fraction, as shown in Table 1. This result can also be visually assessed by comparing the patterns in the two panels of Figure 3. It means that nAbs that are able to bind the spike protein in its closed conformation mostly contribute to the neutralizing activity of convalescent plasma and drive the immune response.

**Table 1:** Average characteristics of the four classes of nAbs computed from the 83 complexes of the $D_{\mathrm{nAb}\text{-}\mathrm{spike}}$ dataset. The steric clash scores (in number of clashing atom pairs N${}_{\mathrm{atoms}}$) are given for the nAb-spike protein complexes in closed, 1-open and 3-open trimer conformations. The angiotensinconverting enzyme 2 (ACE2) overlap is computed as the percentage of RBD residues that bind ACE2 and overlap with a given epitope, on average for each class of nAbs. The Pearson correlation coefficient *r* is computed between the predicted change in binding affinity upon mutation (ΔΔ*G_b_*) (see Section 2.4) on the set of the 20 most frequent receptor binding domain (RBD) variants observed in the circulating viral strains [25] and averaged over the nAb complexes of the different classes, and the measured variant escape fractions from 10 COVID-19 patient plasma [26] and from 23 healthy individuals vaccinated with mRNA-1273 [27].

	NTD	RBD-Closed	RBD-LowClash	RBD-HighClash
〈clashscore〉 in closed state (N${}_{\mathrm{atoms}}$)	9	58	1412	3080
〈clashscore〉 in 1-open state (N${}_{\mathrm{atoms}}$)	23	1	0	947
〈clashscore〉 in 3-open state (N${}_{\mathrm{atoms}}$)	35	30	45	319
〈ACE2overlap〉 (%)	0.0	36.0	72.8	3.7
$r(\Delta \Delta G_{b}$-escape fraction COVID-19)	-	0.64	0.24	0.15
*p*-value		0.003	0.32	0.52
$r(\Delta \Delta G_{b}$-escape fraction mRNA-1273)	-	0.20	0.59	0.18
*p*-value		0.39	0.005	0.45

We calculated the overlap between the residues of the RBD that bind to ACE2 (Section 2.2) and the epitopes of each of the three RBD-targeting nAb classes. We found that the fraction of ACE2 binding residues that overlaps with nAb epitope residues is different for each of the three classes (see Figure 2, green heatmap). Indeed, as shown in Table 1, the epitopes of the RBD-Closed and Open-LowClash classes overlap with ACE2 binding residues by 34% and 72% on the average, respectively, while the epitopes of the RBD-Open-HighClash class basically do not overlap with ACE2 binding residues. This indicates that the ability of nAbs to bind the spike protein in its closed conformation is probably more important than their overlap with ACE2 binding residues.

This result can be considered surprising since nAbs that best mimic ACE2 by binding to a very large portion of the ACE2-RBD interface have been suggested to have the most effective neutralization strategy [29] and are therefore expected to dominate the immune response. Our results as well as others [6,30] rather suggest that the nAb ability to bind the closed conformation of the spike protein, thus blocking its transition to an open state and locking the closed pre-fusion conformation, is a more important feature for having highly neutralizing nAbs dominating the immune response.

We also investigated the contribution of the three RBD-targeting nAb classes to the neutralizing activity of plasma from 23 healthy individuals vaccinated with mRNA-1273 [27]. The correlation between the predicted $\Delta \Delta G_{b}$ caused by the set of 20 most frequent variants in the RBD on the nAb-spike protein affinity and the escape fraction from plasma of vaccinated patients is reported in Table 1. It indicates that the nAb class elicited after mRNA-1273 vaccination is mainly the RBD-Open-LowClash class, while nAbs that are able to bind the closed conformation or belong to the RBD-Open-HighClash class do not appear to contribute significantly.

We thus found different results for COVID-19 patients and for vaccinated individuals: the former seem to preferentially elicit nAbs from the Closed class and the latter, from the RBD-Open-LowClash class. Note that nAbs produced upon COVID-19 infection and after vaccination have already been suggested to target different epitopes [27], even though other analyses indicate that this is not the case [31]. Whether and why the nAbs differ is thus left as a challenging open question. Note that this apparent contradiction can be resolved by noting that the dominant nAb class depends on the infecting strain, as shown by the results of Table 2, and thus also on the immunogen used in the vaccine. Moreover, it could be argued that the spike proteins in the viral envelope and those included in a vaccine have different preferences for the open and closed conformations, and thus could elicit different nAb classes.

### 3.5. Circulating Strains, nAb Classes and Immune Evasion

Finally, we evaluated the ability of SARS-CoV-2 circulating strains to escape the immune response, in view of understanding and predicting which nAb classes are more affected by emerging variants. For that purpose, we computed the changes in binding affinity, $\Delta \Delta G_{b}^{\mathrm{strain}}$, between the spike protein of the different lineages B.1.1.7 (Alpha), B.1.351 (Beta), B.1.617.2 (Delta), and B.1.1.529 (Omicron) and the nAbs contained in each of the four classes (see Section 2.4). They are reported in Table 2, together with the average escape fractions measured from plasma of COVID-19 patients [26] and of healthy individuals vaccinated with mRNA-1273 [27] (see Section 2.5).

No sign of immune evasion is predicted on the basis of the $\Delta \Delta G_{b}^{\mathrm{strain}}$ values for the Alpha strain. In contrast, both Beta and Delta strains are predicted to escape slightly from immune recognition, and the Omicron strain characterized by a large amount of mutations in its spike protein, to escape even much more strongly. This ranking is in agreement with the escape fractions estimated from plasma of COVID-19 patients and of healthy individuals vaccinated with mRNA-1273 (Table 2). Our results are also globally in agreement with a series of other experimental data [1,32,33,34,35].

Our computational approach also provides information on the type of nAbs from which the strains escape: the Beta strain mainly escapes from the RBD-Open-LowClash class, the Delta strain from the NTD and RBD-Closed classes, and the Omicron strain from all four nAb classes. Moreover, our results indicate that the Omicron strain can potentially drive infection in vaccinated individuals or reinfection in recovered COVID-19 patients, which is confirmed by the epidemiological data of the recent Omicron outbreak [36].

## 4. Discussion

Our computational analysis of 83 non-redundant nAbs targeting the SARS-CoV-2 spike protein trimer confirms and specifies the existence of different classes of nAbs, each driving viral evolution in a different way. Indeed, we found that one specific class is the main driver of the humoral immune response in SARS-CoV-2 infected patients, consisting of nAbs which are able to bind the spike protein trimer in both the open and closed conformations. In contrast, vaccination seems to mainly elicit another class of antibodies that are able to bind the spike protein in the open conformation with one or multiple RBDs in standing-up states.

This interesting finding opens a series of questions and suggestions for the understanding of the antigenic evolution and the design of new therapeutic strategies that are summarized below:

- The spike protein evolution must be analyzed in terms of classes of nAbs that recognize different epitopes [14], but also different conformations of the spike protein.
- It must be noted that nAb-RBD crystal structures and their in vitro binding affinity only give partial indications of the neutralizing activity of nAbs. Indeed, they do not account for the different conformations of the spike protein trimer [37].
- Our results show that nAbs recognizing the RBD in the closed state of the spike protein trimer play a pivotal role in shaping the immune response and are thus important for protection (see also [14,30]). This is in agreement with experimental results suggesting that, in the state preceding ACE2 binding and entrance of the virus into the cells, the spike protein trimer spends the majority of the time in closed conformation [38,39], at least when attached to the virus. This suggests to design highly stable closed spike protein trimers as improved vaccine immunogens [30,39,40].
- The contribution of the different classes of nAbs to the neutralizing activity of polyclonal plasma is challenging to analyze. Indeed, it is highly variable among COVID-19 patients, as shown in Figure 3 and Figure S1 of Supplementary Material, and moreover, it evolves over time [26]. For example, even though the immune response of the majority of infected patients appears to be driven by the RBD-Closed nAb class, for a few of them, it is driven by the RBD-Open-LowClash class.
- We found differences in the dominant nAb classes in SARS-CoV-2 infected patients and vaccinated healthy individuals (Table 2 and Figure S1): the RBD-Closed class for the former and the RBD-Open-LowClash class for the latter. This result could be due to the fact that these two classes are elicited by different spike protein conformations, and that the most frequent conformation adopted by the spike protein could differ according to whether it is administered as a vaccine immunogen or attached to the viral membrane. Indeed, although the spike protein trimer in the viral envelope is known to spend most of the time in closed conformation [38,39], the free spike protein could possibly prefer open conformations.
- The analysis of how the contribution of the different classes of nAbs to the immune response has changed as a result of recently circulating viral strains [1], such as the B.1.617.2 (Delta) or B.1.1.529 (Omicron) strains, is very instructive. Indeed, mutations affecting the affinity of immunodominant nAbs can lead to an enhancement of subdominant nAb contributions and to a complex interplay between different nAbs classes.
- The fact that natural infection and mRNA vaccine elicit a different class of nAb could explain why "hydrid" immunization obtained via the combined action of vaccine injection and natural infection elicits a stronger and more robust immune response [41].

## Figures and Tables

**Table 2:** Average predicted change in binding affinity $\Delta \Delta G_{b}^{\mathrm{strain}}$ (in kcal/mol) between the nAbs contained in the different classes and the spike protein of the SARS-CoV-2 viral strain (column 2–5) (see Section 2.4). The last two columns contain the value of the escape fraction of the viral strains estimated from plasma of COVID-19 patients [26] (column 6) and of healthy individuals vaccinated with mRNA-1273 [27] (column 7) (see Section 2.5). Averaged $\Delta \Delta G_{b}^{\mathrm{strain}}$ values bigger than 3 kcal/mol are in red, between 1 and 3 kcal/mol in light red and lower than 1 kcal/mol in white. Escape fraction values are in red if they are bigger than 30%, in light red if they are between 10% and 30%, and in white below 10%.

	$\Delta \Delta G_{b}^{\mathrm{strain}}$	$\Delta \Delta G_{b}^{\mathrm{strain}}$	$\Delta \Delta G_{b}^{\mathrm{strain}}$	$\Delta \Delta G_{b}^{\mathrm{strain}}$	Escape	Escape
	NTD	RBD-Closed	RBD-Open-	RBD-Open-	COVID-19	Vaccine
	nAbs	nAbs	LowClash nAbs	HighClash nAbs	Plasma	mRNA
B.1.1.7 (Alpha)	0.0	0.0	0.1	0.0	0%	0%
B.1.351 (Beta)	0.8	0.8	1.0	0.2	5%	2%
B.1.617.2 (Delta)	1.2	1.0	0.5	0.4	6%	2%
B.1.1.529 (Omicron)	1.7	4.1	5.1	1.6	42%	10%

## Data Availability

Data on PDB structures used in this paper can be downloaded from our GitHub repository (github.com/3BioCompBio/SARS-CoV-2-Epitope, accessed on 2 December 2021).

## Supplementary Materials

The following supporting information can be downloaded at: https://www.mdpi.com/article/10.3390/ijms23042078/s1.

## Acronyms

**PDB**	Protein Data Bank
**nAbs**	neutralizing antibodies
**RBD**	receptor binding domain
**NTD**	N-terminal domain
**ACE2**	angiotensin-converting enzyme 2
**RSA**	relative solvent accessibility
**ΔΔ*G_b_***	change in binding affinity upon mutation
**RMSD**	root mean square deviation

## References

[1] Harvey W.T., Carabelli A.M., Jackson B., Gupta R.K., Thomson E.C., Harrison E.M., Ludden C., Reeve R., Rambaut A., Peacock S.J. (2021). SARS-CoV-2 variants, spike mutations and immune escape. Nat. Rev. Microbiol..

[2] Wintjens R., Bifani A.M., Bifani P. (2020). Impact of glycan cloud on the B-cell epitope prediction of SARS-CoV-2 Spike protein. NPJ Vaccines.

[3] Dai L., Gao G.F. (2021). Viral targets for vaccines against COVID-19. Nat. Rev. Immunol..

[4] Gaebler C., Wang Z., Lorenzi J.C., Muecksch F., Finkin S., Tokuyama M., Cho A., Jankovic M., Schaefer-Babajew D., Oliveira T.Y. (2021). Evolution of antibody immunity to SARS-CoV-2. Nature.

[5] Post N., Eddy D., Huntley C., van Schalkwyk M.C., Shrotri M., Leeman D., Rigby S., Williams S.V., Bermingham W.H., Kellam P. (2020). Antibody response to SARS-CoV-2 infection in humans: A systematic review. PLoS ONE.

[6] Barnes C.O., Jette C.A., Abernathy M.E., Dam K.M.A., Esswein S.R., Gristick H.B., Malyutin A.G., Sharaf N.G., Huey-Tubman K.E., Lee Y.E. (2020). SARS-CoV-2 neutralizing antibody structures inform therapeutic strategies. Nature.

[7] Robbiani D.F., Gaebler C., Muecksch F., Lorenzi J.C., Wang Z., Cho A., Agudelo M., Barnes C.O., Gazumyan A., Finkin S. (2020). Convergent antibody responses to SARS-CoV-2 in convalescent individuals. Nature.

[8] McCallum M., De Marco A., Lempp F.A., Tortorici M.A., Pinto D., Walls A.C., Beltramello M., Chen A., Liu Z., Zatta F. (2021). N-terminal domain antigenic mapping reveals a site of vulnerability for SARS-CoV-2. Cell.

[9] Watanabe Y., Allen J.D., Wrapp D., McLellan J.S., Crispin M. (2020). Site-specific glycan analysis of the SARS-CoV-2 spike. Science.

[10] Henderson R., Edwards R.J., Mansouri K., Janowska K., Stalls V., Gobeil S.M., Kopp M., Li D., Parks R., Hsu A.L. (2020). Controlling the SARS-CoV-2 spike glycoprotein conformation. Nat. Struct. Mol. Biol..

[11] Focosi D., Maggi F., Franchini M., McConnell S., Casadevall A. (2022). Analysis of Immune Escape Variants from Antibody-Based Therapeutics against COVID-19: A Systematic Review. Int. J. Mol. Sci..

[12] Pucci F., Rooman M. (2021). Prediction and Evolution of the Molecular Fitness of SARS-CoV-2 Variants: Introducing SpikePro. Viruses.

[13] Hastie K.M., Li H., Bedinger D., Schendel S.L., Dennison S.M., Li K., Rayaprolu V., Yu X., Mann C., Zandonatti M. (2021). Defining variant-resistant epitopes targeted by SARS-CoV-2 antibodies: A global consortium study. Science.

[14] Greaney A.J., Starr T.N., Barnes C.O., Weisblum Y., Schmidt F., Caskey M., Gaebler C., Cho A., Agudelo M., Finkin S. (2021). Mapping mutations to the SARS-CoV-2 RBD that escape binding by different classes of antibodies. Nat. Commun..

[15] Lu M., Uchil P.D., Li W., Terry D.S., Gorman J., Zhang B., Zhou T., Ding S., Liu L., Ho D.D. (2021). Real-Time Conformational Dynamics of SARS-CoV-2 Spikes on Virus Particles. Biophys. J..

[16] Raybould M.I., Kovaltsuk A., Marks C., Deane C.M. (2021). CoV-AbDab: The coronavirus antibody database. Bioinformatics.

[17] Berman H.M., Westbrook J., Feng Z., Gilliland G., Bhat T.N., Weissig H., Shindyalov I.N., Bourne P.E. (2000). The protein data bank. Nucleic Acids Res..

[18] Li W., Godzik A. (2006). CD-HIT: A fast program for clustering and comparing large sets of protein or nucleotide sequences. Bioinformatics.

[19] Dalkas G.A., Teheux F., Kwasigroch J.M., Rooman M. (2014). Cation–*π*, amino–*π*, *π*–*π*, and H-bond interactions stabilize antigen–antibody interfaces. Proteins Struct. Funct. Bioinform..

[20] Lv Z., Deng Y.Q., Ye Q., Cao L., Sun C.Y., Fan C., Huang W., Sun S., Sun Y., Zhu L. (2020). Structural basis for neutralization of SARS-CoV-2 and SARS-CoV by a potent therapeutic antibody. Science.

[21] Walls A.C., Park Y.J., Tortorici M.A., Wall A., McGuire A.T., Veesler D. (2020). Structure, Function, and Antigenicity of the SARS-CoV-2 Spike Glycoprotein. Cell.

[22] DeLano W.L. (2002). Pymol: An open-source molecular graphics tool. CCP4 Newsl. Protein Crystallogr..

[23] Williams C.J., Headd J.J., Moriarty N.W., Prisant M.G., Videau L.L., Deis L.N., Verma V., Keedy D.A., Hintze B.J., Chen V.B. (2018). MolProbity: More and better reference data for improved all-atom structure validation. Protein Sci..

[24] Dehouck Y., Kwasigroch J.M., Rooman M., Gilis D. (2013). BeAtMuSiC: Prediction of changes in protein–protein binding affinity on mutations. Nucleic Acids Res..

[25] Shu Y., McCauley J. (2017). GISAID: Global initiative on sharing all influenza data–from vision to reality. Eurosurveillance.

[26] Greaney A.J., Loes A.N., Crawford K.H., Starr T.N., Malone K.D., Chu H.Y., Bloom J.D. (2021). Comprehensive mapping of mutations in the SARS-CoV-2 receptor-binding domain that affect recognition by polyclonal human plasma antibodies. Cell Host Microbe.

[27] Greaney A.J., Loes A.N., Gentles L.E., Crawford K.H., Starr T.N., Malone K.D., Chu H.Y., Bloom J.D. (2021). Antibodies elicited by mRNA-1273 vaccination bind more broadly to the receptor binding domain than do those from SARS-CoV-2 infection. Sci. Transl. Med..

[28] Tina K., Bhadra R., Srinivasan N. (2007). PIC: Protein interactions calculator. Nucleic Acids Res..

[29] Ge J., Wang R., Ju B., Zhang Q., Sun J., Chen P., Zhang S., Tian Y., Shan S., Cheng L. (2021). Antibody neutralization of SARS-CoV-2 through ACE2 receptor mimicry. Nat. Commun..

[30] Carnell G.W., Ciazynska K.A., Wells D.A., Xiong X., Aguinam E.T., McLaughlin S.H., Mallery D., Ebrahimi S., Ceron-Gutierrez L., Asbach B. (2021). SARS-CoV-2 spike protein stabilized in the closed state induces potent neutralizing responses. J. Virol..

[31] Wang Z., Schmidt F., Weisblum Y., Muecksch F., Barnes C.O., Finkin S., Schaefer-Babajew D., Cipolla M., Gaebler C., Lieberman J.A. (2021). mRNA vaccine-elicited antibodies to SARS-CoV-2 and circulating variants. Nature.

[32] Planas D., Veyer D., Baidaliuk A., Staropoli I., Guivel-Benhassine F., Rajah M.M., Planchais C., Porrot F., Robillard N., Puech J. (2021). Reduced sensitivity of SARS-CoV-2 variant Delta to antibody neutralization. Nature.

[33] Cao Y., Wang J., Jian F., Xiao T., Song W., Yisimayi A., Huang W., Li Q., Wang P., An R. (2021). Omicron escapes the majority of existing SARS-CoV-2 neutralizing antibodies. Nature.

[34] Liu L., Iketani S., Guo Y., Chan J.F.W., Wang M., Liu L., Luo Y., Chu H., Huang Y., Nair M.S. (2021). Striking Antibody Evasion Manifested by the Omicron Variant of SARS-CoV-2. Nature.

[35] Planas D., Saunders N., Maes P., Benhassine F.G., Planchais C., Porrot F., Staropoli I., Lemoine F., Pere H., Veyer D. (2021). Considerable escape of SARS-CoV-2 variant Omicron to antibody neutralization. Nature.

[36] Ledford H. (2021). How severe are Omicron infections?. Nature.

[37] Barnes C.O., West A.P., Huey-Tubman K.E., Hoffmann M.A., Sharaf N.G., Hoffman P.R., Koranda N., Gristick H.B., Gaebler C., Muecksch F. (2020). Structures of human antibodies bound to SARS-CoV-2 spike reveal common epitopes and recurrent features of antibodies. Cell.

[38] Ke Z., Oton J., Qu K., Cortese M., Zila V., McKeane L., Nakane T., Zivanov J., Neufeldt C.J., Cerikan B. (2020). Structures and distributions of SARS-CoV-2 spike proteins on intact virions. Nature.

[39] Juraszek J., Rutten L., Blokland S., Bouchier P., Voorzaat R., Ritschel T., Bakkers M.J., Renault L.L., Langedijk J.P. (2021). Stabilizing the closed SARS-CoV-2 spike trimer. Nat. Commun..

[40] Xiong X., Qu K., Ciazynska K.A., Hosmillo M., Carter A.P., Ebrahimi S., Ke Z., Scheres S.H., Bergamaschi L., Grice G.L. (2020). A thermostable, closed SARS-CoV-2 spike protein trimer. Nat. Struct. Mol. Biol..

[41] Bates T.A., McBride S.K., Leier H.C., Guzman G., Lyski Z.L., Schoen D., Winders B., Lee J.Y., Lee D.X., Messer W.B. (2022). Vaccination before or after SARS-CoV-2 infection leads to robust humoral response and antibodies that effectively neutralize variants. Sci. Immunol..

