# Supplementary material for: Analysis of the Neutralizing Activity of Antibodies Targeting Open or Closed SARS-CoV-2 Spike Protein Conformations
Source: Int J Mol Sci. 2022 Feb 14;23(4):2078. doi: 10.3390/ijms23042078 (PMC8876721; doi:10.3390/ijms23042078)
Supplement: Supplementary file 1 [file ijms-23-02078-s001.zip › ijms-1560335-supplementary.pdf]

Supplementary Material

Analysis of the neutralizing activity of antibodies
targeting open or closed SARS-CoV-2 spike protein conformations

Gabriel Cia, Fabrizio Pucci, Marianne Rooman

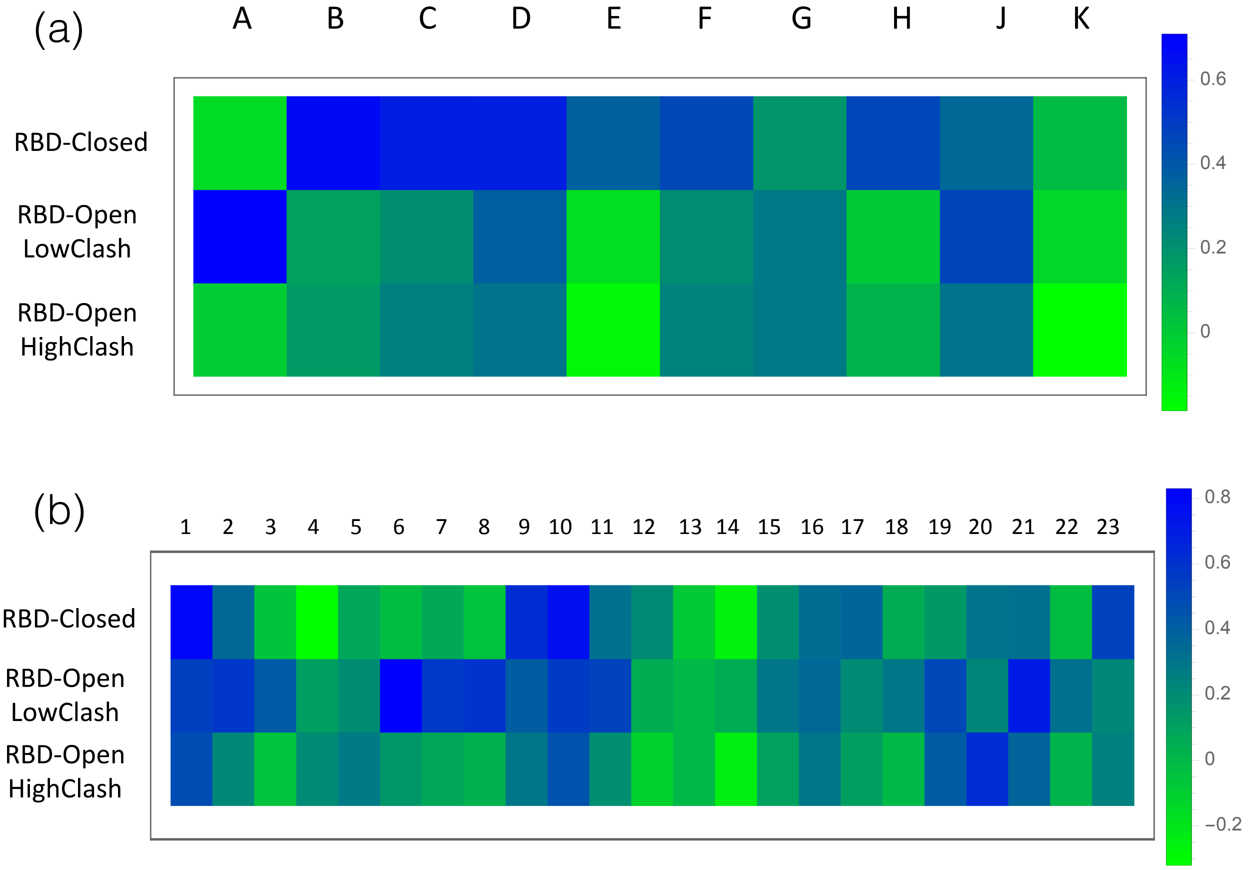

Figure S1. Correlation coefficients between the predicted change in nAb-spike protein binding affinity $\Delta\Delta G_b$ upon mutations (see section 2.4 of the main text) averaged over the nAbs contained in the three RBD-targeting classes (RBD-Closed, RBD-Open-LowClash and RBD-Open-HighClash) and the escape fraction of a set of variants measured from the neutralizing activity of plasma from: (a) 10 SARS-CoV-2 infected patients [A-K] and (b) 23 vaccinated individuals [1-23] (see section 2.5). The mutations analyzed are the set of 20 most frequently observed RBD variants in circulating viral strains (see section 2.4). The color scales are indicated on the right.

Key spike protein residues involved in nAb binding			
RBD-Closed	RBD-Open-LowClash	RBD-Open-HighClash	NTD
449 TYR	417 LYS	369 TYR	144 TYR
452 LEU	421 TYR	372 ALA	147 LYS
456 PHE	455 LEU	374 PHE	154 GLU
484 GLU	456 PHE	377 PHE	156 GLU
486 PHE	486 PHE	378 LYS	158 ARG
489 TYR	489 TYR	380 TYR	246 ARG
490 PHE	505 TYR	384 PRO	248 TYR

Table S1. List of the key spike protein residues involved in nAb binding. Only hydrophobic, ionic, cation-π, and aromatic-aromatic interactions were considered. The values were calculated by running the Protein Interactions Calculator (PIC) webserver on each nAb-RBD or nAb-NTD structure with default parameter values, and selecting the residues that appear most often in interaction with nAb residues.

Key spike protein residues involved in ACE2 binding
403 ARG
417 LYS
484 GLU
486 PHE
489 TYR

Table S2. Key spike protein residues involved in ACE2 binding. Only hydrophobic, ionic, cation-π, and aromatic-aromatic interactions were considered. The values were calculated by running the Protein Interactions Calculator (PIC) webserver on the PDB structure 6M0J with default parameter values.

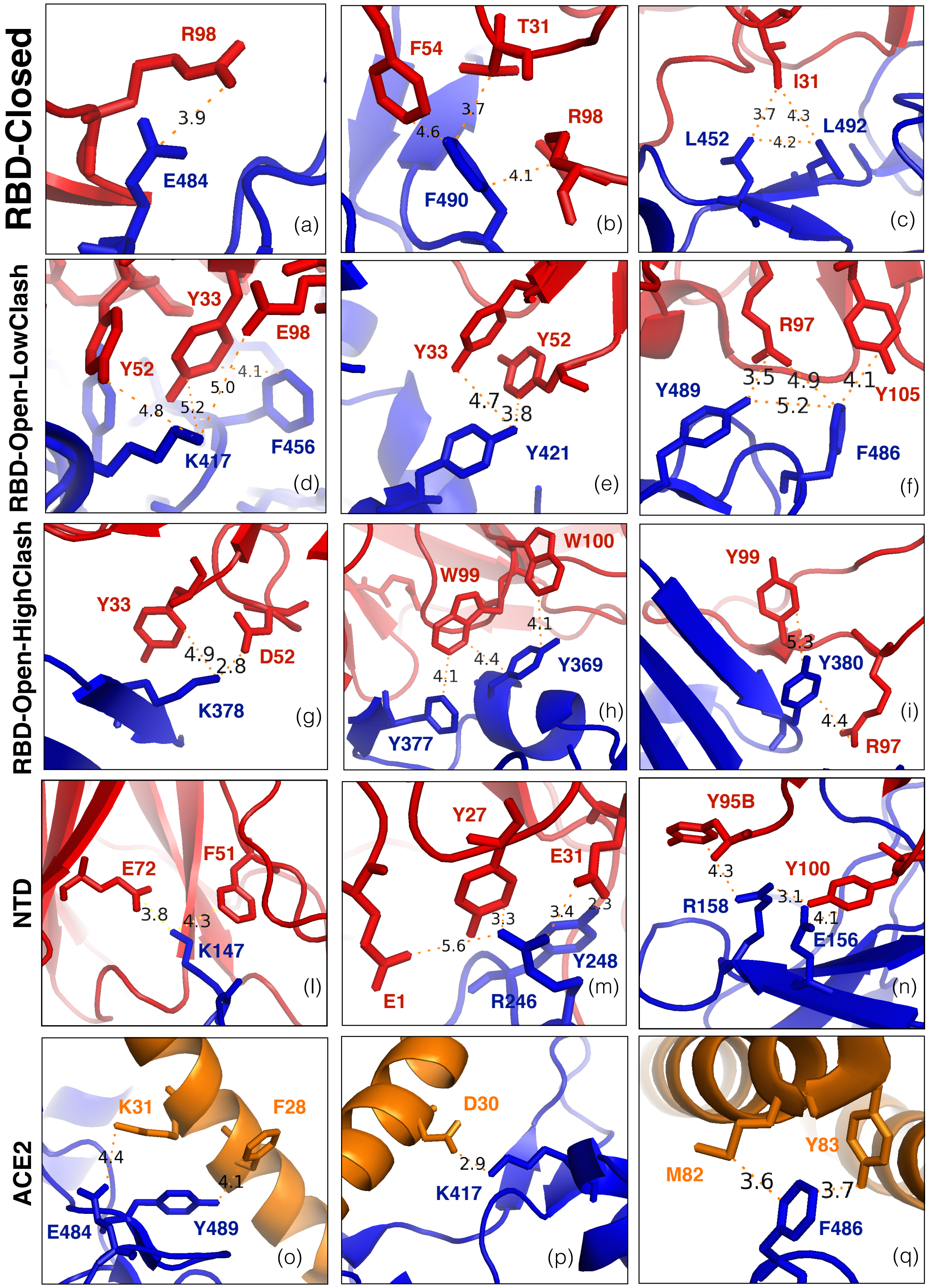

Figure S2. Representation of key spike protein residues and their interactions. (a-n) Residues that are most often involved in spike protein-nAb binding interactions for the four classes of nAbs. The spike protein structure is represented in blue and the nAbs in red. The PDB structures used are (a-c): 7mf1; (d-f) 7km1; (g-h) 7m7b; (i) 7jmw; (l-m) 7c2l; (n) 72le. (o-q) Spike protein-ACE2 binding interactions from PDB structure 6m0j, with spike protein residues depicted in blue and ACE2 residues in orange. Interatomic distances are indicated in Å.
